# Ballet after breast cancer: investigating the feasibility and acceptability of a novel 16-week classical ballet intervention for breast cancer survivors

**DOI:** 10.1007/s00520-022-07420-9

**Published:** 2022-10-27

**Authors:** Eliza R. Macdonald, Briana K. Clifford, David Simar, Rachel E. Ward

**Affiliations:** 1 Department of Exercise Physiology, School of Health Sciences, UNSW Sydney, Sydney, Australia; 2 School of Nursing, Midwifery and Social Work, The University of Queensland (UQ), Brisbane, Australia

**Keywords:** Breast cancer, Dance for health, Classical ballet, Upper-body morbidity, Quality of life, Physical activity

## Abstract

**Purpose:**

The “Ballet after breast cancer” study sought to investigate the feasibility and acceptability of a 16-week classical ballet intervention for breast cancer survivors, delivered face-to-face and/or online.

**Methods:**

Breast cancer survivors were recruited to take part in 2 × 1-h ballet classes per week for 16 weeks. Primary outcomes of feasibility and acceptability were assessed according to rates of enrolment and attendance and participant feedback via questionnaire. Secondary outcomes included quality of life (QOL), upper-body disability, shoulder range of motion (ROM), muscular strength, aerobic capacity, and physical activity levels. Associations between rate of attendance and changes in secondary measures were explored.

**Results:**

Thirty-one participants (62% of eligible individuals) enrolled in the program. Twenty-nine women commenced the intervention [53.3 ± 10.8 years (Mean ± SD)], attending 77.6% [67.6, 87.5] (Mean [95% CI]) of sessions. Based on these rates of enrolment and attendance, and participant feedback, the program was deemed feasible and acceptable to participants. Significant improvements in shoulder ROM and reductions in sedentary behaviour were achieved. Participants also reported improvements in physical capacity and psychological, social, and cognitive wellbeing.

**Conclusions:**

The “Ballet after breast cancer” program, delivered face-to-face and/or online, was feasible and acceptable to breast cancer survivors. Improvements in shoulder ROM achieved doing ballet were pertinent given the adverse effects of upper-body morbidity on breast cancer survivor QOL. Improvements in physical activity behaviour and perceived benefits to wellbeing also support the use of ballet to mitigate QOL impairment after treatment.

**Implications for cancer survivors:**

The physical demands and the fun, creative, and social characteristics of ballet promote improvement across multiple domains of health and wellbeing. Ballet shows promise as an activity to improve QOL and increase long-term engagement in health-promoting physical activity after breast cancer.

**Supplementary Information:**

The online version contains supplementary material available at 10.1007/s00520-022-07420-9.

## Introduction

Breast cancer is the most diagnosed malignancy worldwide. In 2020, female breast cancers represented 11.7% of global cancer diagnoses and accounted for 29% of cancer diagnoses in Australian women [1]. Whilst the likelihood of survival from early breast cancer now exceeds 90% [2], quality of life (QOL) for breast cancer survivors may be undermined by persisting treatment effects, impaired psychosocial wellbeing, and elevated risk of comorbid health conditions and cancer recurrences. It is well established that physical activity can impart numerous benefits to individuals on the cancer continuum, improving physical function, body composition, cardiorespiratory function, metabolic health, and psychosocial wellbeing [3–5]. With side effects including upper-body morbidity (UBM)—such as upper-limb lymphoedema, reduced shoulder range of motion (ROM), and upper-body pain—impaired cardiac function, poor metabolic health, and reduced bone mineral density known to effect breast cancer survivors, the physiological benefits exercise confers are pertinent to this population. Despite the known benefits, reports indicate that 20 to 54% of breast cancer survivors meet current physical activity guidelines [6], with motivation, persisting treatment-related side effects, self-confidence, and access to suitable exercise resources cited as barriers to engagement [7]. Further barriers to exercise faced during the COVID-19 pandemic, such as restricted access to exercise facilities, exercise professionals, and peers to exercise with, may have contributed to the reduction in physical activity and increase in sedentary behaviour observed in general and breast cancer survivor populations at this time [8–10]. Strategies to promote exercise uptake and adherence to help survivors reap the benefits of physical activity during and beyond the COVID-19 era are warranted.

One such strategy is the promotion of dance for health. Dance is a fun, creative, and social activity, which demonstrates potential to improve multiple aspects of health and wellbeing. The positive effects of dance have been observed across healthy and clinical population groups, including cancer patients and survivors [11–18]. Dance has been shown to elicit improvements in cardiometabolic health, cardiorespiratory fitness, muscle strength and power, flexibility, balance, and proprioception [16, 19, 20] as well as mental health and QOL [16], all of which are particularly pertinent after breast cancer treatment.

Classical ballet, like other styles of dance, is an attractive and enjoyable form of physical activity. Ballet has additional characteristics, such as a unique use of the upper-body, which sets it apart as an ideal style of dance for breast cancer survivors. Individuals with treatment-related UBM such as lymphedema, pain, or restricted shoulder ROM are poised to benefit from exercises promoting positive strength/endurance, proprioception, and mobility adaptations to the upper body. The characteristic ballet “port de bras” or “carriage of the arms” (Online Resource 1) through five main upper-body positions calls for postural control, coordination, musculoskeletal endurance, and a large shoulder range of motion (ROM) [21]. Given the potential reductions in QOL associated with UBM, ballet may also contribute to improved QOL by addressing upper-body impairments [22]. Furthermore, ballet is based on a codified movement vocabulary, the basics of which are learned before complex choreographed series are performed [21]. The progressive and repetitive way in which ballet is taught makes it ideal for individuals seeking a graded return to physical activity after breast cancer. Finally, recognising French ballet terminology and being able to recall, anticipate, and execute technical ballet choreography in time with music rely on complex cognitive processing and concentration [23, 24]. This may be of benefit to individuals experiencing cognitive impairment following cancer treatment [25].

Based on findings from other dance genres [12, 14, 15, 18, 26–29] and online exercise interventions delivered to breast cancer survivors [30], ballet shows promise in promoting physical activity adherence and improvements in health and wellbeing in this population. However, no studies have investigated the use of traditional classical ballet, delivered face-to-face or remotely to individuals following completion of primary breast cancer treatment. Therefore, the objective of this study was to investigate the feasibility and acceptability of a novel 16-week classical ballet intervention for breast cancer survivors, delivered face-to-face and/or remotely online. Additionally, the study aimed to identify changes in QOL, physical activity levels, upper-body function, muscular strength, and aerobic capacity of participants after the intervention and identify associations between ballet class attendance and such changes.

## Methods

### Ethics approval

Ethical approval for this study was granted by the UNSW Human Research Ethics Committee (HC200758).

### Study registration

The study was prospectively registered on the Open Science Framework (osf.io/j87vf).

### Sample size and recruitment

As the primary study outcomes were feasibility and acceptability, a priori power calculations based on the secondary outcome QOL were used as a guide for recruitment targets. To detect a moderate effect (*d* = 0.5) of physical activity on QOL, with 85% confidence, a total sample of 31 participants would be necessary. Accounting for 15–20% attrition, a target sample of 40 participants was deemed suitable for the study [12, 18, 28].

### Recruitment

Participants were recruited from the community, via material distributed to breast cancer support groups, organisations, and university social media platforms. Individuals eligible to take part were > 18 years of age, previously diagnosed with stage 0–III breast cancer, and completed primary treatment (surgery, chemotherapy, and/or radiotherapy) > 3 months prior to the intervention. Exclusion criteria were < 18 years of age, currently undergoing active breast cancer treatment (excluding ongoing hormonotherapy or targeted therapy), or had medical conditions and/or physical limitations contraindicating safe participation in dance. Written informed consent was provided by all participants upon enrolment.

### Intervention

The intervention consisted of 1-h classical ballet classes twice per week for 16 weeks and was intended for face-to-face delivery to two intervention groups, successively. Baseline assessments and ballet classes commenced face-to-face for intervention group one (G1) and were converted to online delivery via “Zoom” (Zoom Video Communications, Inc.), from week 6 due to COVID-19 restrictions. Intervention group two (G2) completed all study components online. Secondary outcome assessment methods were modified or substituted for remote delivery, based on evidence demonstrating validity and reliability of online assessments delivered by one assessor [31].

Ballet classes were delivered by an experienced instructor qualified by the Royal Academy of Dance. Each class followed a traditional ballet format, including a general 5-min warm up, 50-min of ballet-based exercise, and 5-min cool down. Classes consisted of stationary exercises at the *barre* (approx. 30 min) involving progressively increasing joint range of motion as in Online Resource 1, followed by a series of dance combinations in the centre of the room (approx. 20 min) usually involving balance training (*adage*), progressing to a series of travelling and turning combinations, and ending with a set of jump exercises (*allegro*). The exercises increased in complexity, intensity, and volume, proportionate to the group’s improvement in physical endurance, skill, coordination, and balance. The program was adjusted for online delivery, accounting for space limitations, by designing exercises that could be performed in smaller spaces, e.g. reduced repetition of travelling steps and vertical rather than horizontal jumping movements. Where necessary, the ballet teacher offered exercise modifications or progressions so all participants could complete a variation of each exercise within the limits of their capability.

### Primary outcome measures

Feasibility was assessed based on rates of enrolment and ballet class attendance. The intervention was deemed feasible if more than 50% of eligible individuals enrolled in the ballet program and if mean rate of class attendance exceeded 75%. Acceptability was assessed using participant responses to a purpose-designed intervention evaluation questionnaire (Online Resource 2). Items referred to the suitability of class frequency and challenge, perceptions of ballet program benefit, ratings of enjoyment, and likelihood of ballet continuation and recommendation to others.

### Secondary outcome measures

#### Quality of life

QOL was assessed using the Functional Assessment of Cancer Therapy, Breast (Lymphedema) (FACT-B + 4) questionnaire (v4) [32]. The questionnaire is validated for use in breast cancer patients/survivors [32] and assesses wellbeing using seven subscales: physical (PWB), social/family (SWB), emotional (EWB), functional (FWB) wellbeing, breast cancer subscale (BCS), and arm symptoms subscale (ARM). Higher scores indicate superior QOL. Permission to use this tool was granted by the Functional Assessment of Chronic Illness Therapy (FACIT) system.

#### Upper-body disability

Self-reported upper-body disability was assessed using the Disability of the Arm Shoulder and Hand (DASH) questionnaire [33]. Questionnaire items relate to limitations in daily activities due to arm, shoulder, and hand morbidity. Low scores (< 15) indicate clinically significant upper-body disability [34].

#### Physical activity levels

Physical activity levels were assessed using the Long International Physical Activity Questionnaire (IPAQ) [35]. Questions relate to the frequency, duration, and intensity of leisure, occupational, transportation, household, and sedentary activities. Questionnaire output included weekly minutes of moderate to vigorous physical activity (MVPA) and average daily sitting time (min/day).

#### Shoulder range of motion

Shoulder ROM data were collected using goniometry during face-to-face assessments (G1 baseline only) or video recordings during online assessments (G1 post-intervention; G2 baseline and post-intervention). ROM data were extracted from videos using “Tracker” software (v5.1.5, Open Source Physics). Participants performed shoulder flexion/extension, abduction, and internal/external rotation in standing. Digital markers were placed on anatomical landmarks, and the protractor function was used to track movement of upper limb markers (humerus, ulnar) relative to the glenohumeral joint centre. For each movement, the average range achieved across three attempts was calculated. To ensure the same fidelity for online as in-person measures, camera placement was replicated at each time point, instructions were provided by the same researcher, and measures were completed in triplicate.

#### Upper-body strength

The maximal push up test using the American College of Sports Medicine (ACSM) protocol was used to assess upper-body strength (G2 only, online) with higher scores indicating superior upper-body strength [5]. Tests were terminated when participants were unable to perform the exercise with correct technique.

#### Lower-body strength

Lower-body strength was assessed using the 30-s chair stand (G2 only, online) [3, 6]. The number of repetitions performed in 30 s was recorded, with higher scores indicating superior lower-body strength.

#### Aerobic capacity

Aerobic capacity was assessed using the 6-min walk test (6MWT) according to the American Thoracic Society (ATS) protocol [36]. G1 completed the 6MWT face-to-face at baseline. Thereafter, (G1 post-intervention; G2 baseline and post-intervention), participants were equipped to conduct the 6MWT from home independently, with the assistance of a friend/family member. Packs containing two field markers, a 20-m rope, and data collection instructions were mailed to each participant prior to their online assessment. Detailed instructions for the conduct of the test were provided by the study coordinator. Greater total walking distance (metres) indicated superior aerobic capacity.

### Statistical analysis

Analysis of the primary outcomes of feasibility and acceptability consisted of mean proportions with a 95% confidence interval using a binomial assumption. Changes in secondary outcome measures including FACT-B + 4, DASH subscale scores, IPAQ questionnaire scores, 6MWT distance, maximal push up score, and 30-s chair stand score were evaluated using paired t-tests. Results are reported as mean change with 95% confidence interval. Linear mixed models were used to evaluate overall changes in shoulder ROM and to determine whether ROM on the affected and unaffected sides changed differentially. ROM change data contained two measures per participant (affected side, unaffected side). “Participant” was included as a random effect to account for dependence. For all secondary analyses, the significance level was set to *α* = 0.05, with *p*-values adjusted for multiple testing. Exploratory regression analyses were performed to evaluate the effect of attendance rate on changes in selected secondary outcome measures. Significance levels were also set to *α* = 0.05, *p*-values unadjusted.

## Results

### Participant characteristics

**Fig. 1:**
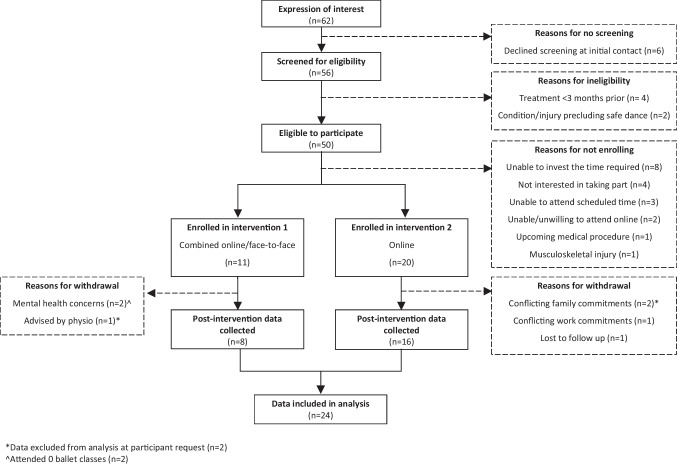
“Ballet after breast cancer” study participation flow

Thirty-one participants enrolled in the study, of which 29 commenced the ballet program and 24 saw it through to completion (Fig. 1). Participants (*n* = 29) were female, aged 53.3 ± 10.8 years (Mean ± SD). Demographic and treatment-related information are provided in Table 1.

**Table 1:** Ballet participant characteristics at baseline

	**Age (years)**		**BMI (kg/m**^**2**^**)**				**Time since diagnosis (months)**			**Time since treatment (months)**			
**n**	29		29				29			29			
**Mean**	53.0		24.9				56.3			52.0			
**SD**	10.6		4.5				47.3			47.4			
**n**		**(%)**		**n**				**(%)**	**n**			**(%)**	
**Cancer type**				**Stage**					**Receptor status**				
Total	28	96.6		Total	29			100	Total	28			96.6
In situ	3	10.3		0–I	4			13.8	ER + /ER-	23/3			79.3/10.3
Invasive	14	48.3		II–III	25			86.2	PR + /PR-	17/5			58.6/17.2
Both	11	37.9							HER2 + /HER2-	12/10			41.3/34.5
Unknown	2	6.9							Unknown	2			6.9
**Breast surgery**				**Axillary surgery**					Reconstruction*				
Total	29	100		Total	22			75.9	Total	14			48.3
Mastectomy	10	34.5		SLNB only	10			34.5	Implant	9			31.0
Double mastectomy	11	37.9		ALND only	6			20.7	Autologous	5			17.2
Breast conserving	8	27.6		Both	6			20.7					
**Chemotherapy**				Radiotherapy*					**Endocrine therapy**				
Total	24	82.8		Total	21			72.4	Total	22			75.9
Neoadjuvant	4	13.8		Breast/chest	21			72.4	Current	16			55.2
Adjuvant	14	48.3		Axilla	8			27.6	Previous	6			20.7
Both	2	6.9		Supraclavicular	6			20.7					
**Targeted/immunotherapy**				**Lymphoedema**					**Peripheral neuropathy**				
Total	12	41.4		Upper-limb	5			17.2	Total		9		31.0
Current	1	3.4							Hands		2		6.9
Previous	11	37.9							Feet		3		10.3
									Both		3		10.3

^*^Not mutually exclusive

*BMI* body mass index, *ALND* axillary lymph node dissection, *SLNB* sentinel lymph node biopsy, *ER* + */ER − *oestrogen receptor positive/negative, *PR* + */PR − *progesterone receptor positive/negative, *HER-2* + */HER-2 − *human epidermal growth factor receptor 2 positive/negative. Presented as *n* (% of total)

### Feasibility and acceptability

Sixty-two percent of eligible individuals enrolled in the study, exceeding the 50% feasibility threshold. The study cohort attended an average of 77.6% [67.6, 87.5] (Mean [95% CI]) of scheduled ballet classes, thus exceeding the feasibility threshold of 75% attendance. G1 and G2 attended 88.3% [78.0, 98.5] and 73.0% [59.5, 86.6] of sessions, respectively.

According to evaluation responses (*Participant quotations italicised*), 80.9% of participants reported the class frequency as “the right amount” and necessary to “*maintain momentum*” and achieve meaningful improvements in skill and physical fitness (Online Resource 2). Some considered one class per week a more realistic commitment long term, due to family and work demands. The level of challenge of the classes was rated “The right level of challenge” by 90.5% of participants. The exercises were “*not so difficult that [they] didn’t want to go back for the next class*” and progressive so that one could be challenged “*a little bit more each time and gradually and logically build strength*”. The classes met expectations for 87.5% of participants, and only somewhat met expectations for 12.5% participants, all of whom were in G2. These participants indicated that online classes did not facilitate expected levels of social engagement, and exercises were less free flowing and physically demanding than anticipated: “*[I] have done many dance classes over the years (ballroom, salsa, swing and flamenco) and was expecting these ballet classes to be a similar level of challenge. However, the classes didn’t challenge me and I didn’t feel I was exercising*”.

The majority (87.5%) of participants rated enjoyment and perceived benefit of ballet classes as ≥ 4/5. The classes were enjoyed as an opportunity to “*learn a completely new skill and new movements*” that were creative and expressive, “*without any pressure to be perfect*”. Dancing was a way of “*exercising without realising it*”, and participants valued “*option[s] for variation in ability levels*”. Participants described improvements in body awareness, fitness, strength, flexibility, balance, and posture. Positive changes in self-esteem, mood, stress levels, and mental focus were reported. In participants with existing musculoskeletal pain, ballet contributed positively to pain management and improved confidence in performing movements they “*didn’t think currently possible*”. Eighty percent of participants were extremely likely to recommend ballet as a “*fun and challenging way to push your physical and mental limits*” and the “*best and most enjoyable method to rehabilitate after cancer*”. Seventy-five percent of participants were “likely” or “extremely likely” to continue with ballet. However, some reported that future participation would be contingent upon the availability of similar ballet programs, targeted towards “*being a late learner with physical challenges*” after cancer. Accessibility, cost, and scheduling of classes were also cited as considerations for ballet continuation.

### Adverse events

One participant experienced light headedness during a face-to-face class, whilst wearing a surgical mask as a COVID-19 precaution. No other adverse events were reported. Minor exercise modifications were provided for participants with joint discomfort or restricted shoulder ROM, where necessary. For example, holding arms in first (below shoulder height), instead of fifth (overhead) position, or replacing jumps with a Plié (Knee bend) and Relevé (Rise).

### Secondary analyses

Changes in secondary outcome measures are presented in Table 2. QOL was maintained over the course of the intervention, indicated by the absence of significant changes in FACT-B + 4 questionnaire scores. Baseline DASH scores were not indicative of clinically significant upper-body disability. No significant changes were observed in self-reported upper-body disability, including for work and sports/performing arts tasks. Significant reductions in sedentary behaviour were observed with participants reporting a reduction in average daily sitting time of − 54.6 min [− 93.1, − 16.2] (*p* = 0.04). Participants increased their weekly duration of MVPA by 279.2 min [− 125.7, 683.9], but this change was not significant (*p* = 0.39). Unadjusted *p*-values indicated significant reductions in upper-body strength (*p* = 0.01) and borderline significant improvements in aerobic capacity (*p* = 0.05). However, following adjustment for multiple testing, no significant changes were observed in aerobic capacity, upper-body strength, or lower-body strength indicated by comparable pre- and post-intervention performance in the 6MWT (*p* = 0.18), maximal push-up test (*p* = 0.06), and 30 s chair stand (*p* = 0.22), respectively. Improvements in shoulder flexion, extension, and abduction were achieved for affected and unaffected sides combined. ROM improved by an average of 6.1° flexion [*t* = 3.74, *df* = 23, 95% CI = 2.86, 9.42, *p* = 0.001], 5.8° extension [*t* = 4.15, *df* = 23.06, 95% CI = 3.01, 8.58, *p* < 0.001], and 6.8° abduction [*t* = 2.96, *df* = 23, 95% CI = 2.19, 11.31, *p* = 0.007] (Table 3). The mean change in shoulder ROM did not differ significantly between affected and unaffected sides for flexion, extension, or abduction.

**Table 2:** Changes in secondary outcome measures

	Pre-intervention					Post-intervention								Change					
	*n* (%)	Mean	95% CI		*n* (%)		Mean	95% CI		*n* (%)		Mean	95% CI			*t*-value	df	*p*-value	
																		Raw	Adj^a^
QOL (FACT-B + 4)																			
Total	29 (100)	110.08	104.69	115.48	24 (82.7)		111.55	104.98	118.12	24 (82.7)		1.22	− 3.26		5.7	0.56	23	0.578	0.692
Physical wellbeing	29 (100)	22.76	21.41	21.11	24 (82.7)		22.96	21.34	24.58	24 (82.7)		0.17	− 0.93		1.26	0.31	23	0.756	0.822
Social wellbeing	29 (100)	21.31	19.67	22.96	24 (82.7)		20.78	18.73	22.83	24 (82.7)		− 0.31	− 1.99		1.38	− 0.38	23	0.709	0.806
Emotional wellbeing	29 (100)	21.36	20.02	22.69	24 (82.7)		22.26	20.89,	23.63	24 (82.7)		0.68	− 0.47		1.83	1.22	23	0.234	0.415
Functional wellbeing	29 (100)	19.69	18.44	20.94	24 (82.7)		19.75	17.99	21.5	24 (82.7)		− 0.4	− 1.64		1.56	− 0.05	23	0.957	0.958
Breast cancer subscale	29 (100)	24.96	22.7	27.21	24 (82.7)		25.79	23.32	28.28	24 (82.7)		0.72	− 1.41		2.86	0.7	23	0.489	0.679
Arm symptom subscale	29 (100)	15.39	13.79	16.99	24 (82.7)		16.46	14.01	18.11	24 (82.7)		1.06	− 0.19		2.32	1.74	23	*0.095*	0.216
Upper-body disability (DASH)																			
Symptoms/disability	29 (100)	37.67	33.81	41.5	24 (82.7)		35.76	31.69	39.8	24 (82.7)		1.63	− 0.96		4.22	1.3	23	0.205	0.394
Work	24 (82.7)	29.69	24.63	34.8	19 (65.6)		26.64	20.39	32.9	17 (58.6)		2.21	− 2.87		7.28	0.92	16	0.307	0.544
Sport/performing arts	9 (31.0)	29.17	16.23	42.1	10 (34.4)		34.38	18.85	49.9	6 (20.7)		− 3.13	− 14.67		8.42	− 0.69	5	0.518	0.682
Physical activity levels (IPAQ)																			
MVPA/week (min)^b^	29 (100)	973.92	615.07	1332.17	24 (82.7)		1025.45	501.12	1549.78	24 (82.7)		259.83	− 136.28		655.94	1.36	23	0.188	0.394
Sitting/day (min)	29 (100)	349.8	289.7	410	24 (82.7)		309.11	253.2	365	24 (82.7)		− 54.64	− 93.11		− 16.17	− 2.94	23	0.007*	0.044*
Aerobic capacity																			
6MWT distance (m)	28 (96.5)	570.48	517.8	623.2	24 (82.7)		613.42	553.32	673.52	24 (82.7)		42.51	− 0.09		85.12	2.06	23	*0.05*	0.179
Lower-body strength^c^																			
30-s chair stand (reps)	19 (65.6)	12.53	11.19	13.9	16 (55.2)		13.56	11.31	15.8	16 (55.2)		1.44	− 0.14		3.02	1.94	15	*0.072*	0.216
Upper-body strength^c,d^																			
Maximal push up (reps)	17 (58.6)	10.18	− 3.34	23.7	14 (48.3)		7	2.38	11.6	14 (48.3)		− 3.64	0.99		6.29	2.97	13	0.011*	*0.055*

^a^Adjusted for multiple testing

^b^Includes walking and MVPA in occupational, home, transportation, and leisure domains

^c^Intervention group two only, due to COVID-19 necessitated changes in assessment methods

^d^*n* = 2 participants unable to complete push up test due to (1) recent abdominal surgery, (2) shoulder instability; sample size presented as *n* (% total)

^*^Statistically significant, *p* < 0.05

**Table 3:** Linear mixed models evaluating change in shoulder ROM

	Mean change, sides^a^ combined (°)	95% CI		t-score (df)	*p*-value		Difference in change between sides^a^ (°)	95% CI		t-score (df)	*p*-value	
					Raw	Adj^b^					Raw	Adj^b^
Flexion	6.14	2.86	9.42	3.74(23)	0.001*	0.013*	− 0.31	− 5.09	4.49	− 0.13(26.83)	0.089	0.216
Extension	5.79	3.01	8.58	4.15(23.06)	< 0.001*	0.009*	− 1.42	− 6.56	3.58	− 0.56(29.28)	0.581	0.692
Abduction	6.75	2.19	11.31	2.96(23)	0.007*	0.044 *	− 4.91	− 13.07	3.66	− 1.18(28.74)	0.249	0.415
Internal rotation	− 8.51	− 18.08	1.06	− 1.78(22.13)	0.089	0.216	0.17	− 5.95	6.39	0.05(21.86)	0.958	0.958
External rotation	4.44	0.66	8.13	2.39(19.37)	0.027*	0.114	3.18	− 5.95	6.39	0.96(23.84)	0.347	0.542

^a^Affected/unaffected sides

^b^Adjusted for multiple testing

^*^Statistically significant, *p* < 0.05

### Exploratory analyses

Exploratory analyses using univariate linear regression provided no evidence to suggest that rate of attendance to the ballet intervention was associated with reductions in sitting time [*t* = 0.535, *df* = 22, 95% CI =  − 1.79, 3.04, *p* = 0.598], improvements in upper-body strength [*t* = 0.96, *df* = 12, 95% CI =  − 0.07, 0.20, *p* = 0.356], or shoulder flexion [*t* =  − 0.12, *df* = 22, 95% CI =  − 0.22, 0.19, *p* = 0.906], extension [*t* = 1.30, *df* = 21.62, 95% CI =  − 0.06, 0.27, *p* = 0.209], or abduction [*t* = 0.33, *df* = 22, 95% CI =  − 0.23, 0.33, *p* = 0.747].

## Discussion

The “Ballet after breast cancer” study aimed to investigate the feasibility and acceptability of ballet for breast cancer survivors and identify changes in objective physical capacity and self-reported outcomes. Based on rates of enrolment and attendance, and participant evaluation, the program was deemed feasible and acceptable. Class attendance rates and participant feedback related to the benefits and suitability of dance were comparable to those reported in other studies of dance post-cancer [12, 14, 17, 37]. As in previous interventions assessing the feasibility of Hawaiian Hula dance [12] and Ballroom dance [17] after cancer treatment, participants of the present study were inclined to recommend ballet to others or express interest in ongoing participation.

Previously, dance interventions for individuals after cancer have produced positive changes in self-reported outcomes, such as QOL [12, 14, 15, 18, 37–39], fatigue [14, 15, 37], physical activity [12, 18], and psychological wellbeing [14, 15, 27, 37, 39], as well as objective outcomes including cardiorespiratory fitness [18, 28, 38], body composition/anthropometry [12, 28, 38], and muscular strength [28, 38]. The perceived improvements in physical capacity, mood, confidence, and cognition reported by participants in this study endorse ballet as another activity with benefits spanning multiple aspects of wellbeing for cancer survivors. Similar health benefits of participation in dance have also been reported for other clinical populations, including Parkinson’s disease, cerebral palsy, stroke, multiple sclerosis, and Alzheimer’s disease [16]. The health effects of classical ballet have been less frequently studied than other dance genres, but positive effects of ballet have been demonstrated in Parkinson’s disease, cerebral palsy, and multiple sclerosis [40]. The present study is the first to report significant, quantifiable improvements in shoulder ROM following a dance intervention (excluding dance movement therapy) for breast cancer survivors. Studies of other dance styles have reported insignificant changes in ROM [38, 41] or have not provided data to quantify described improvements in ROM [27]. Given the prevalence of treatment-related UBM—including impaired shoulder ROM—and the risk of impaired QOL associated with upper-body symptoms and activity limitations [22], this finding is highly pertinent, supporting ballet as an ideal dance style/ activity to minimise the burden of UBM after breast cancer.

The large, but statistically insignificant change in self-reported MVPA should be noted. Participants recorded an average of four additional hours of weekly MVPA, including walking and moderate to vigorous intensity household, transportation, occupational, and leisure activities post-intervention. This may be attributed to the atypically high volume of home maintenance activities (e.g. moving house) reported post-intervention and atypically low baseline MVPA levels due to COVID-19 restrictions.

One feature distinguishing the present study from previous dance interventions was the online delivery. Some aspects of the group class experience were lost by moving online, and this may account in part for the lack of substantial changes in outcome measures, for example, the opportunity to form relationships with other class participants. Previous literature has identified this as a key contributor to the psychosocial benefits of taking part in a group/partnered dance intervention, but this was diminished in the current study because of the online delivery [15, 17, 27]. In addition, due to the space limitations of attending classes from home and the reduced capacity for monitoring participants, the intended intensity and technical correctness of ballet exercises may not have been achieved, contributing to reduced physiological adaptation. The present study was not designed to determine the effectiveness of classical ballet and was thus unable to demonstrate that online delivery attenuated changes in secondary outcome measures. However, findings from another exercise intervention for breast cancer survivors conducted during the COVID-19 pandemic suggests that this might be a reasonable assumption [30]. According to Winters-Stone et al. (2021), a resistance training intervention delivered online demonstrated inferior effectiveness to the equivalent intervention delivered face-to-face, despite achieving higher rates of adherence and retention, and comparable safety [30].

In lieu of a sufficient sample size and control group, the extent to which changes could be attributed to the intervention was inferred. Exploratory analyses sought to elucidate the effect of rate of attendance on changes in secondary outcomes. This may have led to an over- or underestimation of intervention effectiveness as attendance only accounts for one aspect of program engagement. Other factors, such as exercise compliance and social interaction with classmates, may have contributed to program engagement and the magnitude of changes in secondary outcome measures.

Despite the limitations associated with study design, the “Ballet after breast cancer” program demonstrated many strengths. This was the first program to deliver classical ballet classes following a traditional format, to breast cancer survivors after primary treatment. In spite of the restrictions imposed on the study due to the COVID-19 pandemic, the program demonstrated unanticipated strengths. Online delivery was attractive to individuals unable/unwilling to attend in-person classes for reasons including safety, availability, time commitment, and travel. Furthermore, the sense of routine, social interaction, and regular physical activity the program provided may have curtailed an otherwise significant increase in stress, social isolation, sedentary behaviour, and impaired QOL, as has been observed globally during the pandemic [8, 42]. The potential acute effects of each ballet class should also be considered, in light of suggestions of improvements in positive affect, self-esteem, social/community connectedness, and depressive symptoms following a single session of ballet, jazz, or modern dance attended online during the pandemic [43].

## Conclusions

Classical ballet is a feasible and acceptable mode of activity after breast cancer, leading to qualitative reports of improved physical and psychosocial wellbeing. The “Ballet after breast cancer” study provided preliminary evidence to suggest ballet can contribute to improving shoulder ROM and reducing sedentary activity. Delivering the program online helped to overcome several barriers which prevent engagement in physical and social activities after breast cancer, including barriers related to the COVID-19 pandemic. The findings of this study exposed avenues for investigating the comparative effectiveness of ballet and honing its implementation after breast cancer.

## Supplementary Information

Below is the link to the electronic supplementary material.

Supplementary file1 (DOCX 2400 KB)

Supplementary file2 (DOCX 1852 KB)

## Data Availability

The datasets generated in the present study are available from the corresponding author on reasonable request.
