# Supplementary material for: Ballet after breast cancer: investigating the feasibility and acceptability of a novel 16-week classical ballet intervention for breast cancer survivors
Source: Support Care Cancer. 2022 Oct 27;30(12):9909–19. doi: 10.1007/s00520-022-07420-9 (PMC9607692; doi:10.1007/s00520-022-07420-9)
Supplement: Supplementary file 1 — Supplementary file1 (DOCX 2400 KB) [file 520_2022_7420_MOESM1_ESM.docx]

Ballet after breast cancer: Investigating the feasibility and acceptability of a novel 16-week classical ballet intervention for breast cancer survivors

Eliza R. Macdonald, Dr Briana K. Clifford, A/Prof. David Simar, and Dr Rachel E. Ward.

Online resource 1

Online resource 1, Fig. 1. Online ballet classes: “Port de bras” (“carriage of the arms”) performed at the barre, requiring large shoulder ROM.


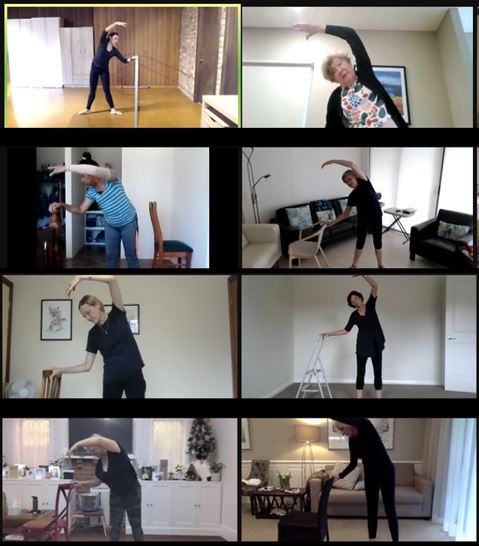

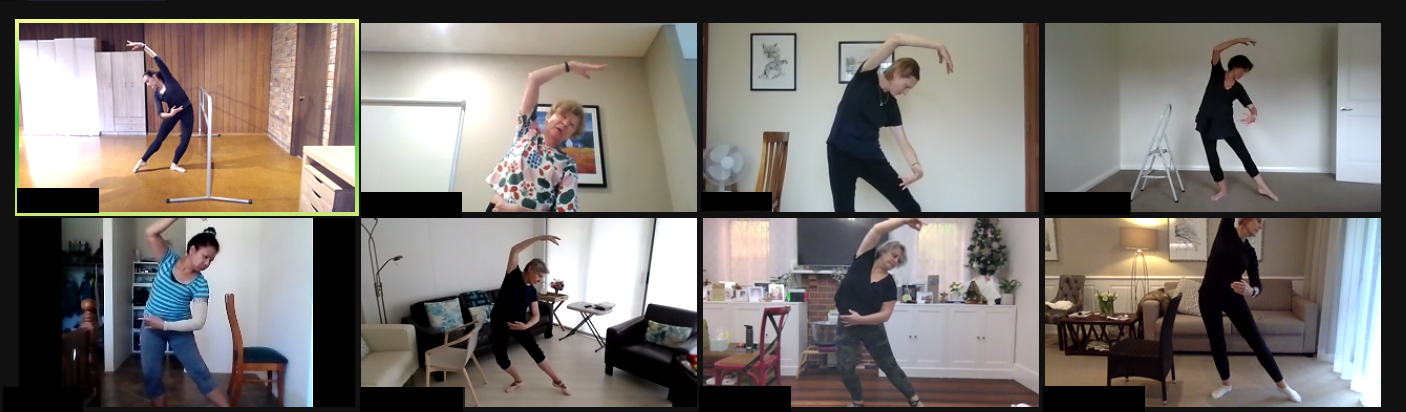

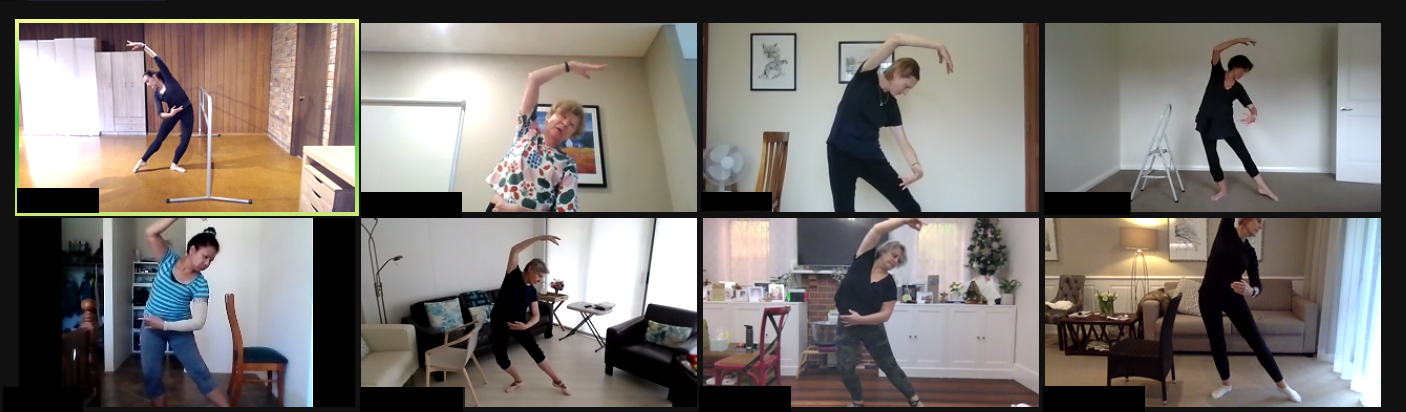


*Online resource 1, Fig. 2 . Online ballet classes: “Port de bras” progression performed away from the barre, requiring increased balance and large shoulder ROM*
