# Supplementary material for: Ballet after breast cancer: investigating the feasibility and acceptability of a novel 16-week classical ballet intervention for breast cancer survivors
Source: Support Care Cancer. 2022 Oct 27;30(12):9909–19. doi: 10.1007/s00520-022-07420-9 (PMC9607692; doi:10.1007/s00520-022-07420-9)

Ballet after breast cancer: Investigating the feasibility and acceptability of a novel 16-week classical ballet intervention for breast cancer survivors

Eliza R. Macdonald, Dr Briana K. Clifford, A/Prof. David Simar, and Dr Rachel E. Ward.

Online resource 2

*
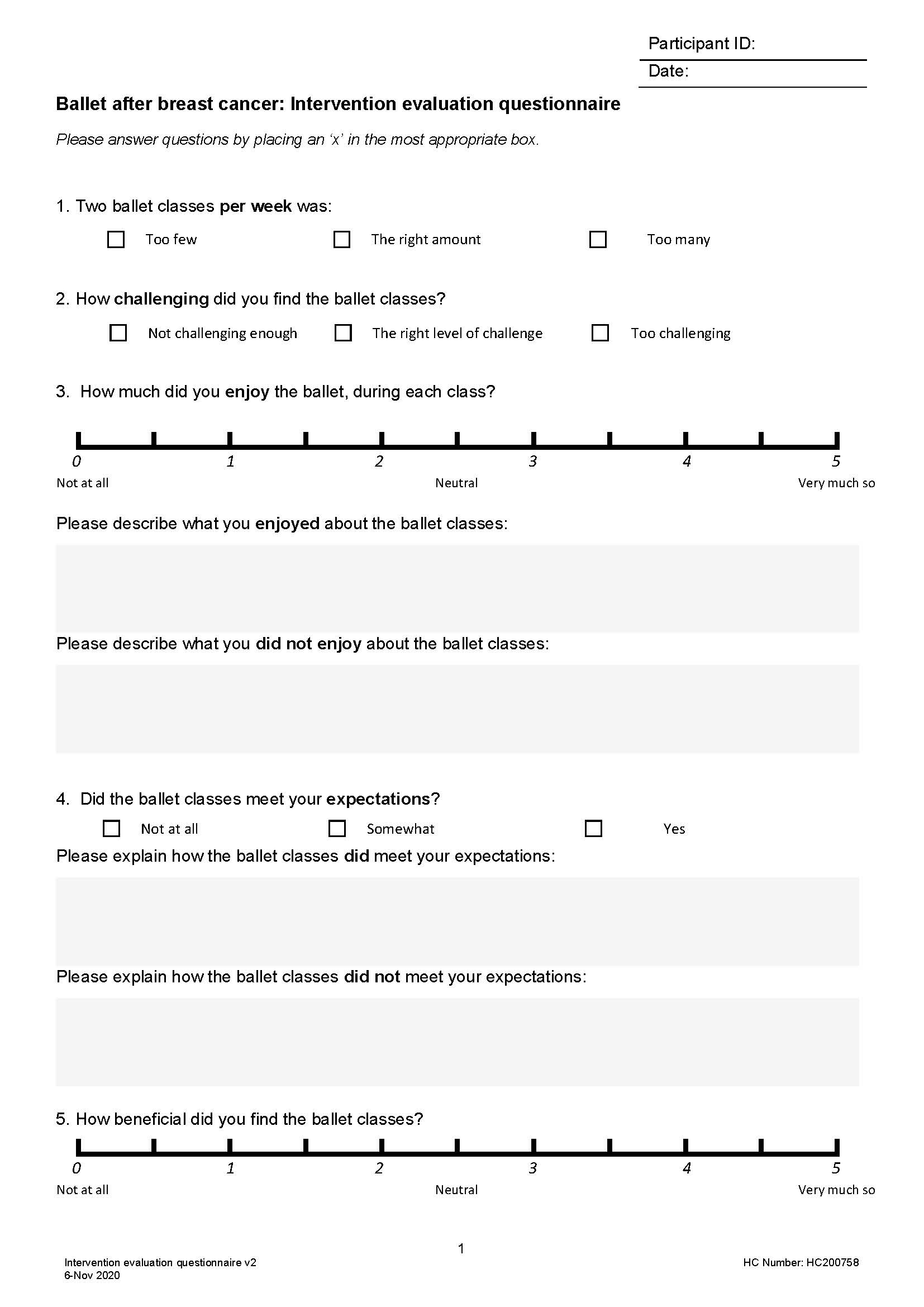
Online resource 2, Supplementary file 1.*
Ballet after breast cancer: Intervention evaluation questionnaire


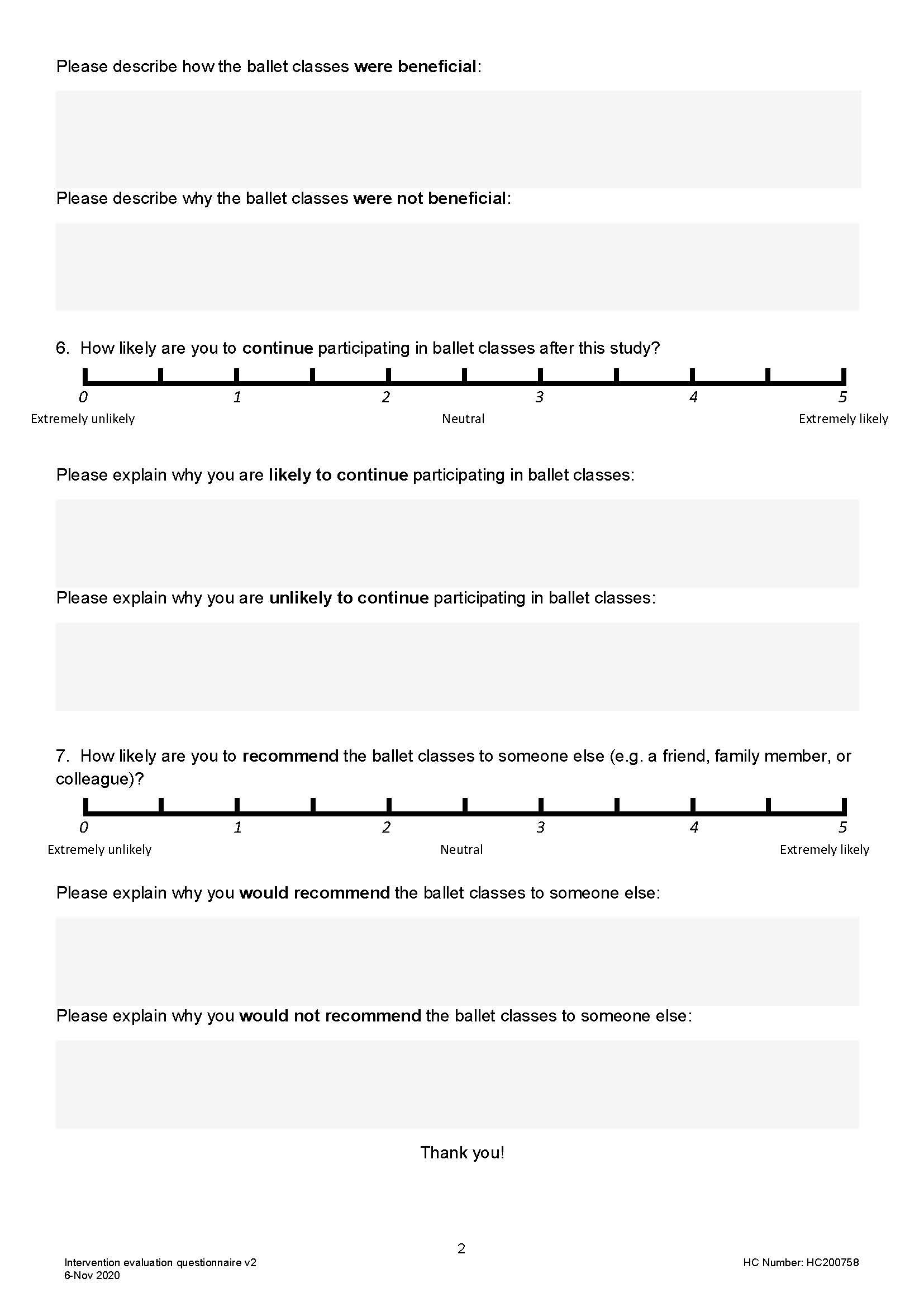


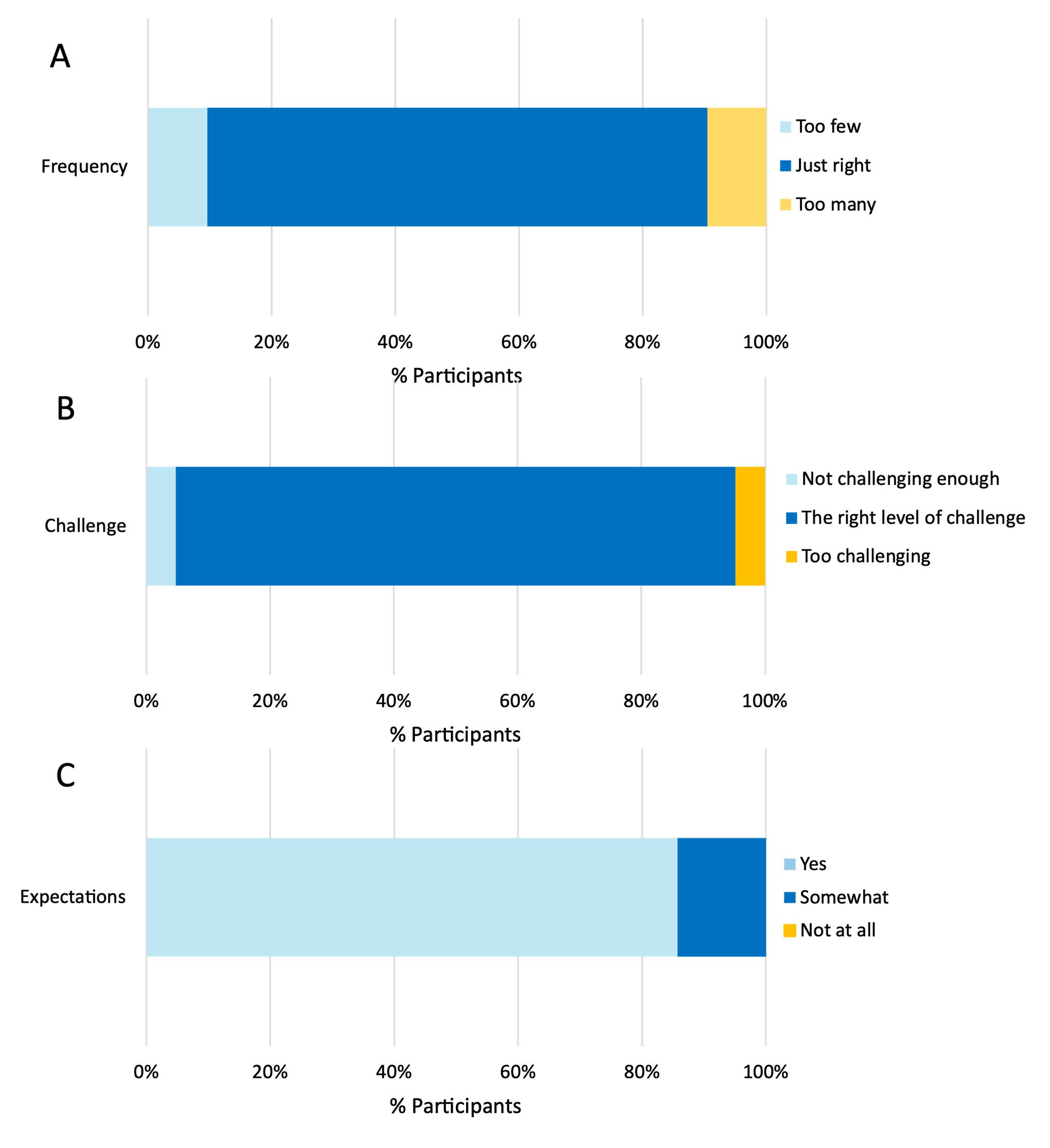


Online resource 2, Fig. 1. Participant evaluation responses regarding the A) suitability of class frequency; B) suitability of the level of challenge of the ballet exercises; C) extent to which ballet classes met expectations.

The right amount

Online resource 2, Fig. 3. Participant evaluation responses regarding their A) Enjoyment of the ballet classes; B) Perceived benefit from the ballet classes; C) Likelihood of continuation of ballet classes; D) Likelihood of recommending ballet to others.


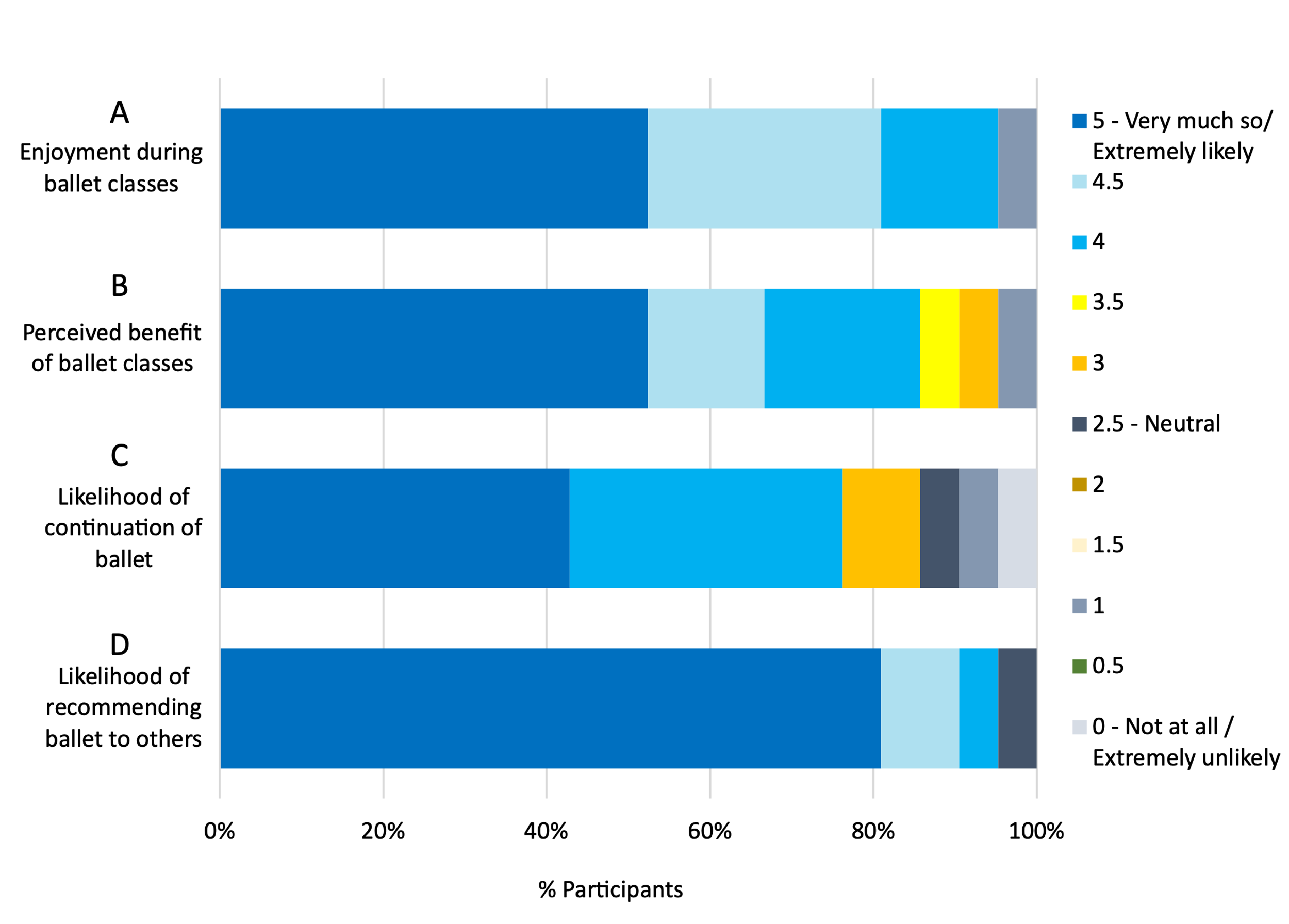

Supplement: Supplementary file 2 — Supplementary file2 (DOCX 1852 KB) [file 520_2022_7420_MOESM2_ESM.docx]
